# Redefining diagnosis-related groups (DRGs) for palliative care – a cross-sectional study in two German centres

**DOI:** 10.1186/s12904-018-0307-3

**Published:** 2018-04-05

**Authors:** Matthias Vogl, Eva Schildmann, Reiner Leidl, Farina Hodiamont, Helen Kalies, Bernd Oliver Maier, Marcus Schlemmer, Susanne Roller, Claudia Bausewein

**Affiliations:** 1 Helmholtz Zentrum Munich, German Research Center for Environmental Health, Institute of Health Economics and Health Care Management, Munich, Germany; 2 Ludwig-Maximilians-Universitaet Munich, Munich School of Management, Institute of Health Economics and Health Care Management & Munich Centre of Health Sciences, Munich, Germany; 3 Munich University Hospital, Department of Palliative Medicine, Ludwig-Maiximilians-Universitaet Munich, Marchioninistr. 15, 81377 Munich, Germany; 4 St. Josephs-Hospital, Department of Palliative Medicine and Interdisciplinary Oncology, Wiesbaden, Germany; 5 Krankenhaus Barmherzige Brüder Munich, Department of Palliative Medicine, Munich, Germany

**Keywords:** Diagnosis related groups, DRG, Costs and cost analysis, Reimbursement, Palliative care, Palliative care funding

## Abstract

**Background:**

Hospital costs and cost drivers in palliative care are poorly analysed. It remains unknown whether current German Diagnosis-Related Groups, mainly relying on main diagnosis or procedure, reproduce costs adequately. The aim of this study was therefore to analyse costs and reimbursement for inpatient palliative care and to identify relevant cost drivers.

**Methods:**

Two-center, standardised micro-costing approach with patient-level cost calculations and analysis of the reimbursement situation for patients receiving palliative care at two German hospitals (7/2012–12/2013). Data were analysed for the total group receiving hospital care covering, but not exclusively, palliative care (group A) and the subgroup receiving palliative care only (group B). Patient and care characteristics predictive of inpatient costs of palliative care were derived by generalised linear models and investigated by classification and regression tree analysis.

**Results:**

Between 7/2012 and 12/2013, 2151 patients received care in the two hospitals including, but not exclusively, on the PCUs (group A). In 2013, 784 patients received care on the two PCUs only (group B). Mean total costs per case were € 7392 (SD 7897) (group A) and € 5763 (SD 3664) (group B), mean total reimbursement per case € 5155 (SD 6347) (group A) and € 4278 (SD 2194) (group B). For group A/B on the ward, 58%/67% of the overall costs and 48%/53%, 65%/82% and 64%/72% of costs for nursing, physicians and infrastructure were reimbursed, respectively. Main diagnosis did not significantly influence costs. However, duration of palliative care and total length of stay were (related to the cost calculation method) identified as significant cost drivers.

**Conclusions:**

Related to the cost calculation method, total length of stay and duration of palliative care were identified as significant cost drivers. In contrast, main diagnosis did not reflect costs. In addition, results show that reimbursement within the German Diagnosis-Related Groups system does not reproduce the costs adequately, but causes a financing gap for inpatient palliative care.

**Electronic supplementary material:**

The online version of this article (10.1186/s12904-018-0307-3) contains supplementary material, which is available to authorized users.

## Background

Patients in palliative care (PC) usually suffer from cancer or advanced neurological, cardiac, pulmonary or renal disease, with a highly individual care utilization [1]. Needs of patients and their family members are diverse. They include relief of symptoms, psychosocial and spiritual as well as practical support [2, 3] – care taking dimensions which need to be reflected by reimbursement calculation. After its introduction in 2003, it has continuously been discussed whether the German Diagnosis-Related Groups (G-DRG) system is an appropriate payment scheme for hospital palliative care units (PCUs) [4, 5]. PC focuses on specific patient and family needs regardless of the underlying diagnosis. In contrast, patient classification by the DRG system mainly relies on main diagnosis or procedures.

In Australia, where the G-DRG system is derived from, [6] “acute care” (treatment driven primarily by patient’s diagnosis) is distinguished from “subacute care” (treatment driven primarily by patient’s functional status and quality of life) [7–9]. It is acknowledged that “sub-acute care” episodes - including PC - are not adequately classified by DRGs and require a different classification approach [10, 11]. A large Australian study demonstrated that the complexity of the patients’ situation, reflected by factors such as phase of illness (stable, unstable, deteriorating, terminal), functional status, severity of symptoms and age, best predicts the resource use and cost in PC, together with the model of PC in the ambulatory setting (multidisciplinary or nursing or medical therapy only) [1, 9]. Consequently, Australia has implemented a special PC reimbursement system outside the DRG-system, based on these factors [7].

Hospital treatment costs and related cost drivers in PC are poorly analysed [12]. It remains unknown whether current G-DRGs, merely accounting for main diagnosis or procedure and developed for acute inpatient care episodes, reproduce costs in PC in Germany adequately. Specifically, it is unknown to which extent, in which direction and in which categories PC costs deviate from the DRG reimbursement.

The aim of this study therefore was to analyse inpatient PC costs and reimbursement, and to identify patient and care characteristics predictive of case costs.

## Methods

### Study design, setting and costing

PC costs were analysed in a cross-sectional, two-center approach. Data were acquired from two German hospitals for all patients who received care on their PCUs from July 2012 to December 2013 (“group A”): a university hospital providing 10 PCU beds, and a non-university hospital providing PCU 30 beds. Data for group A, including length of stay (LOS) data, refer to the complete stay of the patients in the hospital, not only encompassing the stay on the PCU, but also possible days on a normal ward or the intensive care unit. A subgroup of cases with a PCU stay only in 2013 (group B) was also analysed. For 2012, data were unavailable to identify this subgroup.

Clinical and socio-demographic data were acquired from hospital medical records, inpatient costs on patient level from the hospitals’ costing systems. Costs had been calculated using the InEK costing scheme (Institute for the Hospital Remuneration System) [13, 14]. Investment costs for PC were not calculated [15]. The InEK costing scheme is an activity-based full cost approach which has become a generally accepted national costing standard [13]. Each cost center and cost category in a hospital is represented in a cost-matrix, where a cost classifier is defined for each cost module (combination of cost center and cost category). For example, costs of physicians (cost category) on the PCU are allocated to patients based on their LOS (cost classifier). PC is part of the normal ward cost center within this calculation, and is not specifically reflected by any subgrouping. For most cost categories on the ward, LOS is used as the cost classifier to allocate costs to patients. For diagnostic or procedural cost centers such as operating room, radiology or laboratory tests, point systems, actual duration or surgery time are used as cost classifiers. This kind of activity-based micro costing is used in several countries alike and allows comparison of costs between health care systems [16–18].

In the G-DRG-system, a procedure code for specialist palliative care (SPC) was introduced, defined by criteria for SPC acknowledgement and the duration of SPC provision (< 7 days, 7–13 days, 14 to 20 days or > 20 days). A supplementary fee is provided for SPC > 7 days, increasing with the duration of SPC. We used the actual reimbursement of each case, including supplementary fees (e.g. for duration of SPC and for time-consuming nursing care), and distributed it based on the national average cost matrix for the respective DRG, to analyse differences between costs and reimbursement (profit) at the cost-module level of the cost-matrix. We set profit distribution in relation to LOS.

### Statistical analysis

Cost and reimbursement data are given as mean and standard deviation (SD). Spearman’s rank correlation was used to test the relationship of current cost classifiers and costs. The relationship of LOS and costs is visualized by a scatterplot with a linear and LOESS fit line (locally weighted scatterplot smoothing). LOESS was chosen to give an intuitive, optical view on trends in the scatterplot. Therefore, the best fitting models on localized subsets of points in the plot are used instead of a global function, to generate an overall fit line [19].

**Fig. 1:**
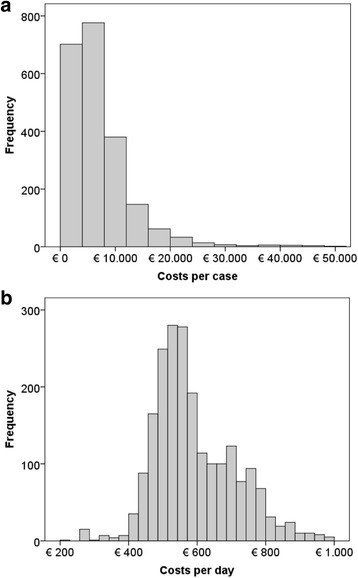
Cost distribution per case (**a**) and per day (**b**) for group A (total group, *n* = 2151)

To analyse the effects of key cost drivers, such as clinical and socio-demographic factors, on case costs, we used a generalized linear model (GLM) with gamma distribution and log link function. A simple ordinary least squares (OLS) model would have required Gaussian distribution, and was thus not able to process the right skewed (*Kolmogorov-Smirnov-Test*, *p* < 0.000) case cost data (Fig. 1) [20]. The GLM suits best for the integration of high cost cases to a single model and can reduce heteroscedasticity of OLS residuals [21, 22]. The GLM model provides relative cost differences for each key cost driver via exponentiated coefficients. For the nearly normal distributed case costs per day, we used OLS regression. Covariates included in regression analyses (independent of being significant in bivariate analysis) were location, age, gender, discharge reason, kind of supplementary fees for SPC, other supplementary fees (e.g. for very costly drugs), LOS, Major diagnostic category (MDC), number of side diagnoses and number of operation and procedure codes. Main diagnosis and DRG were not considered in regression analysis due to too many values (degrees of freedom) to be reported. To test in multivariate analyses whether the current DRG differentiation by main diagnosis is discriminative for costs, MDC was used as a proxy for main diagnosis and DRG. A *p*-value of < 0.05 was considered significant. Statistical analyses were performed with SPSS 22.

To test a DRG-grouping based on current cost classifiers and determine cut-off values, we used classification and regression tree (CART) analysis. CART is a decision tool that has been used in defining the Australian case-mix classification for PC [9] and is able to maximise homogeneity within DRGs by defining cut-off values for the strongest predictors of costs, which serve as the splitting variables [23].

The study was approved by the ethics committee of Ludwig-Maximilians-Universität Munich (reference number: 24–15).

## Results

### Sample characteristics

Between July 2012 and December 2013, 2151 patients received care in the two hospitals including, but not exclusively, on the PCUs (group A). In 2013, 784 patients received care on the two PCUs only (group B). Both groups were similar regarding age, gender, and proportions of malignant disease. Proportions of patients who died on the unit, of patients who received SPC for at least 7 days and the mean number of secondary diagnoses were also similar between groups. Mean LOS was 12 ± 10 days for group A and 10 ± 6 days for group B (Table 1).

**Table 1:** Sample characteristics

Group		A = total group		B = “pall. Care only” group	
Number of patients		*n* = 2151		*n* = 784	
Variable		n; mean	%; SD	n; mean	%; SD
Location	BBM	1555	72.3%	659	84.1%
	LMU	596	27.7%	125	15.9%
Year	2012	1070	49.7%		
	2013	1081	50.3%	784	100%
Gender	male	1031	47.9%	350	44.6%
	female	1120	52.1%	434	55.9%
MDC	respiratory system	331	15.4%	99	12.6%
	hepatobiliary system and pancreas	309	14.4%	123	15.7%
	digestive system	296	13.8%	127	12.2%
	nervous system	288	13.4%	91	11.6%
	other	262	12.2%	116	14.8%
	skin, subcutaneous tissue & breast	191	8.9%	60	7.7%
	kidney and urinary tract	126	5.9%	42	5.4%
	female reproductive system	120	5.6%	35	4.5%
	poorly differentiated neoplasms	119	5.5%	43	5.5%
	male reproductive system	109	5.1%	48	6.1%
Discharge	death	1299	60.4%	455	58%
	home	590	27.4%	237	30.2%
	hospice	159	7.4%	55	7.0%
	nursing home	55	2.6%	25	3.2%
	other hospital	30	1.4%	6	0.8%
	other	18	0.8%	6	0.8%
Cancer	yes	1497	69.6%	549	70%
	no	654	30.4%	235	30%
Kind of suppl. Fee for SPC	palliative care ≤6 days (=no fee)	732	34.0%	240	30.6%
	palliative care 7–13 days	797	37.1%	313	39.9%
	palliative care 14–20 days	466	21.7%	181	23.1%
	palliative care ≥21 days	156	7.3%	50	6.4%
age (years)		69.8	12.37	70.7	11.8
length of stay (days)		12.2	10.10	9.8	6.3
number of secondary diagnoses		14.3	6.50	14.3	5.8
number of operation/procedure codes		4.7	5.28	3.3	1.7

BBM (Hospital Barmherzige Brüder München), LMU (University Hospital Munich), MDC (main Diagnostic Category)

### Costs and reimbursement

For group A, mean total costs per case were €7392 (SD 7897, range 134–132769). Mean total reimbursement per case, including supplementary fees, was €5155 (SD 6347), creating a total financing gap of €2237 per case. Mean case costs per day were €578 (SD 143). 84% of the costs per case occurred on the ward, 10% were related to diagnostics and therapy and 4% to intensive care. However, only 70% of reimbursement was distributed to the cost centre “ward” (i.e. all wards, including the PCU). 62% of the overall financing gap was related to nursing, 15% to physicians, and 35% to medical and non-medical infrastructure on the cost centre “ward”, respectively. For laboratory tests, reimbursement was more than double the costs (270%). The financing gap on the cost centre “ward” (all wards including the PCU) was €2599. For this cost centre, 58% of the overall costs, and 48%, 65% and 64% of costs for nursing, physicians and (medical and non-medical) infrastructure were reimbursed, respectively (Table 2). For group B, i.e. the subgroup of patients with a stay on a PCU only, mean total costs per case were €5763 (SD 3664), mean total reimbursement per case €4278 (SD 2194), creating a total financing gap of €1485. On the PCU, the financing gap was €1767. 67% of overall costs, and 53%, 82%, 72% of costs for nursing, physicians and (medical and non-medical) infrastructure on the PCU were reimbursed, respectively (Table 3). For laboratory tests, more than 7-fold (730%) of the costs were reimbursed.

**Table 2:** Mean costs and reimbursement per case in Euros, separated by cost categories and cost centers for group A (total group, *n* = 2151)

	Physicians	Nursing	Medical/ technical staff	Drugs	Implants/ grafts	Material	^b^Medical infrastructure	^b^Non-medical infrastructure	Sum	%
Cost matrix of palliative care cases										
Ward (including palliative care ward)	982	2658	45	208	0	140	500	1659	6193	83.8%
Intensive care	47	114	1	46	0	27	22	55	312	4.2%
Dialysis	2	7	0	0	0	7	1	2	18	0.2%
Operating room	21	0	23	2	18	22	11	15	112	1.5%
Anesthesia	21	0	14	2	0	5	3	6	51	0.7%
Cardiac diagnostics/therapy	1	0	0	0	1	2	0	0	5	0.1%
Endoscopic diagnostics/therapy	6	0	7	1	2	11	5	5	36	0.5%
Radiology	29	0	36	0	3	21	16	19	125	1.7%
Laboratory tests	4	0	22	47	0	48	4	7	133	1.8%
Further diagnostics/therapy	87	0	170	5	0	16	42	86	406	5.5%
sum	1200	2778	318	313	25	299	604	1855	7392	
%	16.2%	37.6%	4.3%	4.2%	0.3%	4.0%	8.2%	25.1%		
Matrix used for reimbursement^a^										
Ward (including palliative care ward)	638	1276	26	160	0	121	337	1035	3594	69.7%
Intensive care	55	116	2	21	0	23	19	49	286	5.5%
Dialysis	4	11	0	1	0	11	1	3	31	0.6%
Operating room	34	0	27	2	20	28	14	21	146	2.8%
Anesthesia	23	0	14	2	0	5	3	6	53	1.0%
Cardiac diagnostics/therapy	0	0	0	0	1	1	0	0	3	0.1%
Endoscopic diagnostics/therapy	6	0	7	0	0	6	3	5	27	0.5%
Radiology	55	0	65	1	0	19	30	47	218	4.2%
Laboratory tests	32	0	124	18	0	109	18	59	359	7.0%
Further diagnostics/therapy	99	0	183	3	0	20	27	105	437	8.5%
sum	945	1403	450	208	22	343	453	1331	5155	
%	18.3%	27.2%	8.7%	4.0%	0.4%	6.7%	8.8%	25.8%		

^a^overall absolute value represents actual reimbursement, distribution of absolute value represents hypothetical InEK calculation based on study patients

^b^medical infrastructure: e.g. pharmacy, hygiene; non-medical infrastructure: e.g. management, energy, laundry

**Table 3:** Mean costs and reimbursement per case in Euros, separated by cost categories and cost centers for group B (“palliative care only” 2013; *n* = 784)

	Physicians	Nursing	Medical/ technical staff	Drugs	Implants/ grafts	Material	^b^Medical infrastructure	^b^Non-medical infrastructure	Sum	%
Cost matrix of palliative care cases										
Ward	782	2465	35	87	0	100	464	1450	5382	93.4%
Intensive care	0	0	0	0	0	0	0	0	0	0.0%
Dialysis	0	0	0	0	0	0	0	0	0	0.0%
Operating room	3	0	4	0	3	3	2	2	17	0.3%
Anesthesia	3	0	2	0	0	1	0	1	7	0.1%
Cardiac diagnostics/therapy	0	0	0	0	0	0	0	0	0	0.0%
Endoscopic diagnostics/therapy	3	0	4	0	0	2	3	2	15	0.3%
Radiology	4	0	5	0	0	2	2	3	15	0.3%
Laboratory tests	1	0	6	8	0	11	1	2	29	0.5%
Further diagnostics/therapy	27	0	148	4	0	12	36	71	298	5.2%
Sum	823	2465	204	100	4	130	507	1531	5763	
%	14.3%	42.8%	3.5%	1.7%	0.1%	2.3%	8.8%	26.6%		
Matrix used for reimbursement^a^										
Ward	643	1295	12	158	0	63	340	1044	3615	84.5%
Intensive care	0	0	0	0	0	0	0	0	0	0.0%
Dialysis	0	0	0	0	0	0	0	0	0	0.0%
Operating room	3	0	2	0	6	1	1	2	18	0.4%
Anesthesia	2	0	1	0	0	0	0	1	5	0.1%
Cardiac diagnostics/therapy	0	0	0	0	0	0	0	0	0	0.0%
Endoscopic diagnostics/therapy	4	0	5	0	0	1	2	3	16	0.4%
Radiology	19	0	20	0	0	3	9	15	69	1.6%
Laboratory tests	18	0	77	4	0	34	11	36	212	5.0%
Further diagnostics/therapy	90	0	142	3	0	8	20	72	343	8.0%
Sum	780	1295	259	166	6	111	383	1172	4278	
%	18.3%	27.2%	8.7%	4.0%	0.4%	6.7%	8.8%	25.8%		

^a^overall absolute value represents actual reimbursement, distribution of absolute value represents hypothetical InEK calculation based on study patients

^b^medical infrastructure: e.g. pharmacy, hygiene; non-medical infrastructure: e.g. management, energy, laundry

**Fig. 2:**
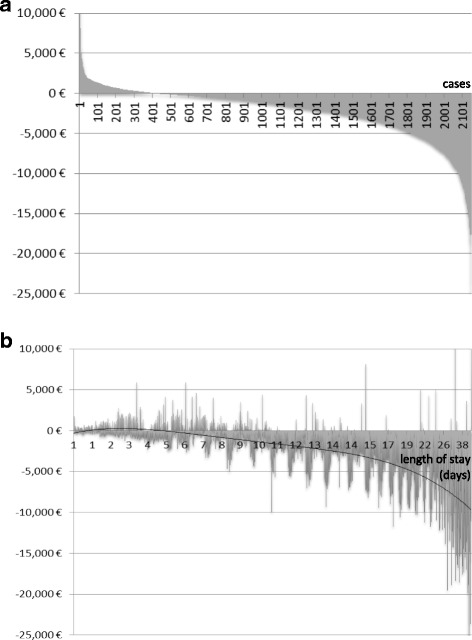
Benefit distribution of cases with highest benefit to highest loss (**a**) and with lowest to highest length of stay (**b**) for group A (*n* = 2151)

Including both standard G-DRG reimbursement and supplementary fees, the benefit distribution of cases with highest profit to highest loss shows that about 21% of all cases generated a profit of on average €1078, while 79% of all cases generated a loss of on average €3086 Euros (Fig. 2a). The benefit distribution according to total LOS shows that for the average case, the break-even point was around 6–9 days, while longer stays caused losses in most cases (Fig. 2b).

### Bi- and multivariate analysis

**Fig. 3:**
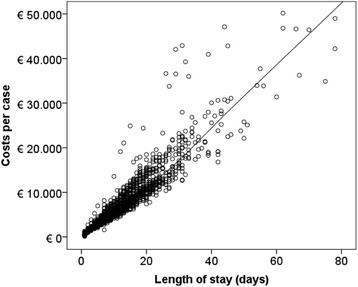
Costs and length of stay per case for group A (*n* = 2151). Notes: Bivariate relationship of length of stay (LOS) with costs. LOESS fit line serves to smooth scatterplot data. R^2^ = 0.78

*Spearman’s* correlation with costs identified total LOS, the kind of supplementary fee for SPC (generally reflecting duration of SPC) and the number of operation and procedure codes (German classification used to encode surgical and medical procedures) as the most relevant factors influencing costs. The number of secondary diagnoses, main diagnosis, DRGs, and MDCs were also significantly associated with costs, but did not correlate highly (see Additional file 1). LOESS fit line analysis confirms total LOS as a good cost driver in the current costing scheme (Fig. 3).

Multivariate analysis showed that total LOS, the kind of supplementary fee for SPC (reflecting the duration of SPC), number of side diagnoses and location influenced costs per case for the total group of patients (group A) and for the patients with purely SPC (group B) (Table 4). Each additional day increased case costs by 5.6% (group A) and 11.3% (group B), and each additional side diagnosis increased costs by 1.5% (group A) and 1.1% (group B). Compared to the shortest duration of SPC, costs increased between 62% and 79% (group A) and between 39% and 91% (group B) dependent on the duration of SPC – with highest costs for 14–20 days SPC. Costs were higher in the university hospital than in the non-university hospital. MDC did not significantly influence costs.

**Table 4:** GLM log link regression on costs per case

Group		A = total group			B = “palliative care only” group		
Number of patients		*n* = 2151			*n* = 784		
Variable		exp(b)	(95% CI)	*p*-value	exp(b)	(95% CI)	*p*-value
Location	BBM	−36.3%	(−39.2%; −33.3%)	**0.000**	−38,0%	(−42,0%; − 33,8%)	**0.000**
	LMU	ref.			ref.		
Gender	male	−4.2%	(−7.3%; −1.0%)	**0.011**	0,9%	(−3,0%; 4,9%)	0.646
	female	ref.			ref.		
Kind of suppl. Fee for SPC	SPC for 7–13 days	62.1%	(55.7%; 68.8%)	**0.000**	39,4%	(17,1%; 66,0%)	**0.000**
	SPC for 14–20 days	79.3%	(69.7%; 89.5%)	**0.000**	90,9%	(66,7%; 118,5%)	**0.000**
	SPC for ≥21 days	69.2%	(55.3%; 84.5%)	**0.000**	60,9%	(45,3%; 78,1%)	**0.000**
	SPC for 0–6 days (=no fee)	ref.			ref.		
Other suppl. Fee	no	−38.4%	(−45.3%; − 30.8%)	**0.000**	−5,8%	(−27,7%; 22,8%)	0.659
	yes	ref.			ref.		
Discharge	home	6.8%	(2.8%; 10.8%)	**0.001**	5,7%	(1,0%; 10,7%)	**0.018**
	to other hospital	3.6%	(−9.0%; 17.8%)	0.595	30,7%	(5,9%; 61,4%)	**0.013**
	to hospice	7.5%	(1.1%; 14.4%)	**0.022**	−0,3%	(−7,7%; 7,6%)	0.935
	to nursing home	8.5%	(−1.6%; 19.7%)	0.101	6,3%	(−4,4%; 18,2%)	0.259
	other	−5.7%	(−20.0%; 11.2%)	0.485	−27,5%	(− 41,0%; −10,8%)	**0.002**
	death	ref.			ref.		
MDC	other	4.6%	(−3.1%; 13.0%)	0.251	−0,2%	(−8,6%; 9,0%)	0.973
	nervous system	9.4%	(1.3%; 18.1%)	**0.022**	7,9%	(−1,1%; 17,7%)	0.086
	respiratory system	2.6%	(−4.8%; 10.5%)	0.499	4,6%	(−3,8%; 13,9%)	0.292
	digestive system	−0.1%	(−7.4%; 7.7%)	0.973	4,7%	(−4,3%; 14,4%)	0.316
	hepatobiliary system & pancreas	3.1%	(−4.4%; 11.1%)	0.431	4,2%	(−4,3%; 13,4%)	0.348
	skin, subcutaneous tissue & breast	−0.8%	(−8.6%; 7.7%)	0.848	0,7%	(−8,6%; 11,1%)	0.882
	kidney and urinary tract	1.3%	(−7.3%; 10.7%)	0.777	0,9%	(−9,1%; 12,1%)	0.861
	male reproductive system	4.4%	(−4.9%; 14.7%)	0.367	−3,7%	(−14,0%; 7,7%)	0.509
	female reproductive system	−0.4%	(−9.1%; 9.1%)	0.924	4,2%	(−6,3%; 15,8%)	0.452
	poorly differentiated neoplasms	ref.			ref.		
Age		0.1%	(0.0%; 0.2%)	0.169	0,0%	(−0,1%; 0,2%)	0.887
Length of stay		5.6%	(5.3%; 6.0%)	**0.000**	11,3%	(10,4%; 12,2%)	**0.000**
No. of side diagnoses		1.5%	(1.1%; 1.8%)	**0.000**	1,1%	(0,6%; 1,5%)	**0.000**
No. of procedure codes		0.0%	(−0.4%; 0.5%)	0.982	1,8%	(0,5%; 3,1%)	**0.008**

BBM (Hospital Barmherzige Brüder München), LMU (University Hospital Munich), MDC (main Diagnostic Category)

Bold numbers: significant results (α-level 0.05)

Linear regression on costs per day showed that location and the number of procedure codes significantly influenced costs per day for group A and B. LOS only influenced costs per day for group A (see Additional file 2).

CART also identified total LOS as the most important cost driver, followed by the kind of supplementary fee for SPC which reflects duration of SPC (only for group A). All other regression variables were no good classifiers in CART (see Additional file 3).

## Discussion

### Cost and reimbursement situation

To our knowledge, this is the first study analysing costs and reimbursement for individual SPC cases. Results show that the current reimbursement system in Germany does not reflect the costs for SPC cases in the hospital. Given the growing evidence that certain interventions or treatments, e.g. laboratory tests or imaging, are less likely after involvement of SPC, over-reimbursement for SPC in a diagnosis-based reimbursement system would also have been conceivable [24, 25]. On the contrary, both absolute and relative differences between overall costs and overall reimbursement in the study sample as well as between costs and reimbursement of cost modules (ward vs. other cost centers, and e.g. nurses versus other cost categories) were large. This applies both for group A (all patients on the PCU, including those treated on other hospital wards before referral to the PCU) and for group B (patients treated on the PCU only). The patients of group B were externally referred, had a shorter LOS, lower costs and smaller differences between costs and reimbursement. The main reason for the smaller financing gap for group B is probably the shorter LOS and the fact, that most costs in PC are attributed to cases via LOS, whereas reimbursement in the DRG system is not mainly influenced by LOS. Thus, our data show that G-DRGs do not reproduce costs for patients treated on the PCU, whether they had a phase of “acute care” on a non-PC ward before referral to the PCU or were treated on the PCU only. As costs or reimbursement for group A could not be differentiated by “acute care phase” or PCU phase, it is not possible to draw any conclusions regarding acute care alone.

Most of the financing gap between costs and reimbursement appeared for nursing costs on the cost centre “ward”, while for laboratory tests, reimbursement was up to 7-fold higher (group B) than the costs. Within reimbursement calculation in the DRG-system, PC patients and non-PC cases are mixed up in a single DRG. As ordinary cases are much more frequent, the more complex and costly PC cases barely influence DRG reimbursement [4]. Within this costing system, the PCU is thus treated like an ordinary ward, and use of resources such as professionals’ time is poorly reflected. Nursing and physician costs, however, are the biggest cost pool for PC, and the ward makes up 84% of overall case costs for group A and 93% for group B, while laboratory costs play a marginal role. The situation is mitigated by supplementary fees for the duration of SPC, otherwise the discrepancy between costs and reimbursement would be even higher.

Most studies on costs or cost-effectiveness in PC focus on specific diseases, [26–28] few report on general inpatient and outpatient PC costs [29–32]. Most work on cost drivers and reimbursement is on the development of the Australian PC reimbursement system outside the DRG-system.[7, 9, 33]. Within the UK, a special reimbursement system for PC patients is currently evolving [34]. This study shows that the German reimbursement system needs changes, too, in order to meet the costs of inpatient PC – for patients treated on non-PC wards and then referred to the PCU as well as for those treated on the PCU only. The current loss-making system may disincentivise hospital palliative care. This is especially problematic, as incentives for SPC at home are lacking, too, and SPC at home is still not available all over the country [35]. For an adequate, needs-oriented care for PC patients, changes to the current reimbursement system in order to achieve adequate reimbursement are essential. In heavily ageing populations, the challenge to adapt the DRG system to more complex needs in a multimorbid, multiply restricted patient population may even extend beyond the scope of PC.

### Redefining DRGs for palliative care

One of our most important findings is that main diagnosis, by which current DRGs are mainly generated, is not an adequate parameter to define costs for PC patients. Significant cost drivers were the kind of supplementary fee for SPC, which reflects the duration of SPC, and total LOS. Both might only be sensitive cost drivers because the current costing system allocates most relevant costs to cases dependent on their LOS. Cost drivers identified in Australia like the complexity of the patient’s situation - which is reflected in the time nurses and physicians spend for the patient – are not assessed in the current system. Actual time spent is used increasingly as a cost classifier in healthcare, and detailed costing systems, so-called time-driven activity-based costing (TDABC), exist to measure costs incurred [13, 36, 37].

There is an ongoing debate on G-DRG’s in PC. The new hospice and palliative care law introduced in Germany in 2015 allows hospitals to leave the DRG system and negotiate day-related fees for PCUs with the sickness funds. From 2017 onwards, individual supplementary fees for SPC hospital advisory teams can be negotiated, which will be standardised for Germany in 2019. To adequately link reimbursement to the complexity of the patients’ situation, we see a need to include the variables used in Australia such as phase of illness, functional status and problem severity [9, 33]. This could be introduced on different levels: 1) Revising the supplementary fee for SPC by inclusion of factors reflecting complexity, 2) Introducing new “DRGs” for PC patients, as already proposed by other authors, [4] separating cases based on complexity of the patients’ situation, and 3) development of an entirely new reimbursement system for PC outside the DRG system. Guided by the Australian and UK experiences, pilot studies are currently underway to develop a casemix classification for German PC, based on complexity of the patients’ situation. On the cost side, elements of TDABC will be used in accordance with the Australian study [9, 13, 36, 37].

Palliative care has been found to be cost-saving compared to usual care, [24, 25, 31, 38–43] and the aging of societies will considerably increase the need of palliative care [44, 45]. An adequate financing system for inpatient palliative care is thus key to a cost-conscious health care system. Hospitals will benefit from adequate reimbursement for PC patients that is calculated from actual PC patients and not mainly from patients in similar DRGs on normal wards. From a long-term policy perspective, this will turn PCUs from loss-making units that have to be cross-subsidised to economically viable units.

### Strengths and limitations and a future perspective

A strength of this study is that we used actual reimbursement of our cases and distributed it on the matrix according to the national calculation dataset, to make cost and reimbursement calculation comparable. A limitation is that we analysed only cases from two centres. These may not be representative of all German PCUs. If they were less efficient than other units, this could have increased the financing gap [13]. In fact, costs were higher in the university hospital than in the non-university hospital, but this may well be explained by the more complex conditions and needs of the patients cared for. To reach a more representative case-mix for PC in Germany, a multi-centre study is necessary. However, in the present study, a large number of cases was analysed, and study patients were treated at a university hospital and a general hospital PCU, well reflecting the German PC situation.

A further limitation is a time gap in the data used, as InEK cost calculations refer to the preceding year. In 2012, a new procedure code was introduced in the G-DRG system, reflecting the duration of SPC on PCUs. The supplementary fee for this was introduced in 2014. Therefore, the reimbursement data used here do not yet entail this new supplementary fee, which may contribute to a reduction of the financing gap in the future. Besides, the G-DRG system is updated annually, which means that every year slightly different base rates and DRG weights are applied. However, as we compare costs and reimbursement on case level, this difference between 2012 and 2013 does not affect the total financing gap. These aspects reflect a general challenge of evaluations of international health care systems, which are continuously changing. Evaluations are only possible with a time lag due to the reasons mentioned above. If the system has changed before the analysis is finished, the question is open once again, whether the current reimbursement system is reflecting the costs.

A general limitation of this study is that all reported costs were calculated based on cost classifiers pre-defined by the InEK costing scheme such as LOS, and that the data do not include the variables identified as significant cost drivers in Australia [9]. However, this cost calculation method is the current national standard in Germany, and thus provides a relevant starting point for analysing the current situation. To better reflect the costs accrued by the care for the individual patients, the resources used by the patients, especially the time spent with the patients by SPC team members, has to be recorded, i.e. a TDABC approach, as done in the Australian study, the ongoing UK study and planned in future studies in Germany [9, 46].

## Conclusions

Main diagnosis, by which current DRGs are mainly generated, is not an adequate parameter to define costs for PC patients. Besides, G-DRGs do not reproduce costs for inpatient PC episodes adequately. Possible reasons for this are 1) the fact that PC patients differ from acute patients on normal wards regarding the complexity of their situation and the care they need, and 2) that the cost classifiers currently used for DRG grouping poorly reflect resource use – especially the use of professionals’ time, which accounts for the biggest cost pool in PC. Studies collecting resource-use based cost data as well as data on the cost drivers identified in Australia are needed as a basis for further development of our costing and reimbursement system for PC. Changes to the current system are crucial to make PCUs economically viable and enable them to provide adequate care to the increasing number of patients in need of PC in the future.

## Additional files

Additional file 1: Spearman’s correlation coefficient on costs per case for group A (*n* = 2151). (DOCX 18 kb)

Additional file 2: Multiple linear regression on costs per day. (DOCX 19 kb)

Additional file 3: Results of the Classification and regression tree analysis (CART) for group A and B. (DOCX 17 kb)

## Abbreviations

BBM: Hospital Barmherzige Brüder München
CART analysis: Classification and regression tree analysis
G-DRG: German Diagnosis-Related groups
GLM: Generalized linear model
InEK: Institute for the Hospital Remuneration System (Institut für das Entgeltsystem im Krankenhaus)
LMU: University Hospital Munich
LOS: Length of stay
MDC: Major diagnostic category
OLS model: Ordinary least squares model
PC: Palliative care
PCU: Palliative care unit
SD: Standard deviation
SPC: Specialist palliative care
