# Supplementary material for: Redefining diagnosis-related groups (DRGs) for palliative care – a cross-sectional study in two German centres
Source: BMC Palliat Care. 2018 Apr 5;17:58. doi: 10.1186/s12904-018-0307-3 (PMC5887171; doi:10.1186/s12904-018-0307-3)
Supplement: Supplementary file 1 — Spearman’s correlation coefficient on costs per case for group A (n = 2151). (DOCX 18 kb) [file 12904_2018_307_MOESM1_ESM.docx]

**Additional file 1: Spearman’s correlation coefficient on costs per case for group A (n = 2151)**

Notes: All correlations are significant at the 0.01 level (2-tailed).
BBM (Hospital Barmherzige Brüder München), LMU (University Hospital Munich), MDC (main Diagnostic Category)
