# Supplementary material for: Redefining diagnosis-related groups (DRGs) for palliative care – a cross-sectional study in two German centres
Source: BMC Palliat Care. 2018 Apr 5;17:58. doi: 10.1186/s12904-018-0307-3 (PMC5887171; doi:10.1186/s12904-018-0307-3)
Supplement: Supplementary file 2 — Multiple linear regression on costs per day. (DOCX 19 kb) [file 12904_2018_307_MOESM2_ESM.docx]

**Additional file 2: Multiple linear regression on costs per day**

| group | |  | | A = total group | | | B = „pall. care only“ group | | |
| --- | --- | --- | --- | --- | --- | --- | --- | --- | --- |
| number of patients | |  | | n = 2151 | | | n = 784 | | |
| variable | |  | | coeff. | SE | p-value | coeff. | SE | p-value |
|  | | intercept | | 607.0 | 30.4 | **0.000** | 597.4 | 95.7 | **0.000** |
| location | | BBM | | -120.7 | 8.2 | **0.000** | -181.3 | 15.8 | **0.000** |
|  | | LMU | | ref. | | | ref. | | |
| gender | | male | | -0.4 | 6.1 | 0.949 | 12.9 | 9.5 | 0.177 |
|  | | female | | ref. | | | ref. | | |
| kind of suppl. fee | | SPC for 7-13 days | | -17.7 | 7.6 | **0.021** | 77.5 | 41.9 | 0.064 |
| for SPC | | SPC for 14-20 days | | -9.4 | 10.3 | 0.361 | 33.7 | 32.5 | 0.299 |
|  | | SPC for ≥ 21 days | | -6.8 | 15.6 | 0.664 | 12.9 | 25.0 | 0.605 |
|  | | SPC for 0-6 days (=no fee) | | ref. | | | ref. | | |
| other suppl. fee | | no | | 76.6 | 21.7 | **0.000** | 20.8 | 65.0 | 0.749 |
|  | | yes | | ref. | | | ref. | | |
| discharge | | home | | -23.2 | 7.0 | **0.001** | -15.9 | 11.3 | 0.160 |
|  | | to other hospital | | -63.6 | 23.8 | **0.008** | -110.2 | 51.5 | **0.032** |
|  | | to hospice | | 9.0 | 11.5 | 0.436 | 8.0 | 18.8 | 0.669 |
|  | | to nursing home | | -7.1 | 18.1 | 0.696 | -3.3 | 26.0 | 0.898 |
|  | | to other hospital | | 44.7 | 30.6 | 0.143 | -43.0 | 51.6 | 0.405 |
|  | | death | | ref. | | | ref. | | |
| MDC | | other | | -11.7 | 14.2 | 0.411 | 7.4 | 21.4 | 0.729 |
|  | | nervous system | | 61.1 | 14.2 | **0.000** | 44.7 | 21.2 | **0.035** |
|  | | respiratory system | | 12.2 | 13.7 | 0.373 | 14.0 | 20.6 | 0.497 |
|  | | digestive system | | -2.3 | 14.0 | 0.870 | -9.2 | 21.8 | 0.671 |
|  | hepatobiliary system & pancreas | | | 8.7 | 13.8 | 0.531 | 4.0 | 20.9 | 0.849 |
|  | skin, subcutaneous tissue & breast | | | 7.5 | 15.2 | 0.619 | 7.8 | 23.9 | 0.743 |
|  | kidney and urinary tract | | | 22.0 | 16.4 | 0.180 | 12.3 | 25.6 | 0.629 |
|  | male reproductive system | | | 4.5 | 17.4 | 0.797 | -5.6 | 27.4 | 0.839 |
|  | female reproductive system | | | 1.8 | 16.8 | 0.913 | -8.3 | 25.9 | 0.748 |
|  | poorly differentiated neoplasms | | | ref. | | | ref. | | |
| age | |  | | 0.3 | 0.2 | 0.184 | 0.6 | 0.4 | 0.106 |
| length of stay | |  | | -1.2 | 0.6 | **0.039** | 0.3 | 1.8 | 0.888 |
| no. of side diagnoses | |  | | -0.2 | 0.6 | 0.683 | 0.6 | 1.1 | 0.617 |
| no. of procedure codes | | |  | -3.6 | 0.9 | **0.000** | 7.5 | 3.1 | **0.017** |

Notes: BBM (Hospital Barmherzige Brüder München), LMU (University Hospital Munich), MDC (main Diagnostic Category)

Bold numbers: significant results (α-level 0.05).
