# Supplementary material for: Redefining diagnosis-related groups (DRGs) for palliative care – a cross-sectional study in two German centres
Source: BMC Palliat Care. 2018 Apr 5;17:58. doi: 10.1186/s12904-018-0307-3 (PMC5887171; doi:10.1186/s12904-018-0307-3)
Supplement: Supplementary file 3 — Results of the Classification and regression tree analysis (CART) for group A and B. (DOCX 17 kb) [file 12904_2018_307_MOESM3_ESM.docx]

**Additional file 3: Results of the Classification and regression tree analysis (CART) for group A and B**

| To determine cut-off values for DRG-grouping, Classification and regression tree analysis (CART) can be used. CART is a non-parametric decision tool, able to handle skewed costing data. | | | | | | |  |  |
| --- | --- | --- | --- | --- | --- | --- | --- | --- |
| It can identify the strongest predictors in regressions by maximizing homogeneity within groups by similarly determining the most favorable cut-off values (LOS and supplementary fee groups in our case) and eliminating variables that do not allow discriminating homogenous groups. | | | | | | |  |  |
|  |  |  |  |  |  |  |  |  |
| **3a) CART analysis group A** |  |  |  |  |  |  |  |  |
| **1. splitting on LOS intervals** | **LOS** | **<7 days** | **7 - 10 days** | **11-14 days** | ***>14 days** | |  |  |
|  | mean costs | 2.144 € | 4.895 € | 7.159 € | 14.434 € | |  |  |
|  | SD | 1.092 € | 1.112 € | 1.637 € | 11.101 € | |  |  |
|  | n | 625 | 461 | 420 | 645 | |  |  |
| ***2. splitting on supplementary fee for SPC groups in the >14 days interval** | **LOS** |  |  |  | ****suppl. fee for 6 -13 days and ≥ 21 days** | *****suppl. fee for 14 - 20 days** |  |  |
|  | mean costs |  |  |  | 17.293 € | 11.601 € |  |  |
|  | SD |  |  |  | 13.582 € | 6.840 € |  |  |
|  | n |  |  |  | 321 | 324 |  |  |
| ** expensive cases due to long intensive care and shorter SPC (suppl. fee for 6 - 13 days) or due to long SPC (≥ 21 days) | | | | | | | | |
| *** cheaper cases due to less intensive care | | |  |  |  |  |  |  |
|  |  |  |  |  |  |  |  |  |
| **3b) CART analysis group B** |  |  |  |  |  |  |  |  |
| **1. splitting on LOS intervals** | **LOS** | **<6 days** | **6 - 10 days** | **>10 days** |  |  |  |  |
|  | mean costs | 1.931 € | 4.836 € | 9.145 € |  |  |  |  |
|  | SD | 915 € | 1.040 € | 3.038 € |  |  |  |  |
|  | n | 223 | 242 | 319 |  |  |  |  |
